# Reproducibility of quantitative real-time PCR assay in microRNA expression profiling and comparison with microarray analysis in narcolepsy

**DOI:** 10.1186/s40064-015-1613-3

**Published:** 2015-12-24

**Authors:** Zhenhua Liu, Liling Yang, Yingzi Zhao, Minglu Tang, Fumin Wang, Xiaoting Wang, Guanzhen Li, Yifeng Du

**Affiliations:** Sleep Medicine Center, Shandong Provincial Hospital Affiliated to Shandong University, 324 Jingwuweiqi Road, Jinan City, 250000 Shandong Province People’s Republic of China; Department of Neurology, Shandong Provincial Hospital Affiliated to Shandong University, 324 Jingwuweiqi Road, Jinan City, 250000 Shandong Province People’s Republic of China

**Keywords:** miRNA, Narcolepsy, Microarray, qRT PCR, Validation

## Abstract

MicroRNAs (miRNAs) have been shown in the pathogenesis of human neurological disorders. The study aims to identify the involvement of miRNAs in the pathophysiology of narcolepsy. Here, we conducted three independent high-throughput analysis of miRNA (miRNA microarray) in peripheral blood from 20 narcolepsy patients who fulfilled the criteria compared to 20 healthy controls with validation experiment using quantitative real-time polymerase chain reaction (real-time PCR) panels. By analyzing 2805 miRNAs in peripheral blood with microarray we identified 128 miRNAs (105 high expression and 23 low expression) that were different in patients with narcolepsy in comparison with healthy control. Then we chose six high expression candidates and six low expression candidates of at least twofold difference and p value < 0.05 to validate the changes in three independent experiments in vitro using real-time PCR. The validation test showed that levels of hsa-mir-1267, hsa-miR-4309, hsa-miR-554, hsa-miR-1272, hsa-miR-4501, hsa-miR-182-3p were higher, whereas the level of hsa-miR-625-5p, hsa-miR-100-5p, hsa-miR-125b-5p, hsa-miR-197-3p, hsa-miR-4522, hsa-miR-493-5p was lower in narcolepsy patients than healthy controls. The levels of 12 miRNAs differed significantly in peripheral blood from narcolepsy patients which suggested that alterations of miRNAs expression may be involved in the pathophysiology of narcolepsy.

## Background

Narcolepsy is a life-long sleep disorder characterized by excessive daytime sleepiness, cataplexy, sleep paralysis, and hypnagogic hallucinations (Thorpy 2015). Usually caused by an interplay of genetic and environmental factors, narcolepsy is a debilitating condition which can affect all aspects of life of which psychosocial functioning and quality are reduced (Zeitzer 2013). Recent studies shows that the impact of sleep disturbance such as narcolepsy is increasingly highlighted on the general outcome of a variety of diseases, like epilepsy, Alzheimer, and Parkinson (Shan et al. 2015; Economou et al. 2012). Usually sleep disorder/disturbance can be hard to diagnose with psychosocial consequences (Espie et al. 2014). Nearly all patients diagnosed with narcolepsy require lifelong treatment. Unfortunately, till now the pathogenetic molecular mechanism of narcolepsy is not yet fully understood, which showed the difficulty in effective prediction, prevention and treatment.

During the past three decades, molecular analysis for disease became more and more important. Especially, microRNAs (miRNAs) are considered to be key regulators and valuable biomarker candidates for various human pathologies. Recently, alterations in expression of miRNAs in both human and animal model have been linked to many forms of disease. miRNAs are a class of 20- to 22-nt, endogenous, noncoding RNAs. These small moleculars usually act as post-transcriptional regulators of gene expression by base pairing with their target messenger RNAs (mRNAs) (Igaz and Igaz 2015; Chen et al. 2015). Some miRNAs have been identified to regulate neuronal processes in the nervous system such as brain morphogenesis, neuronal cell differentiation, and transcription of neuronal-specific genes (Xue et al. 2013). The role of miRNAs in sleep regulation has been studied in experimental animal models. For example, miRNAs expression change in rats’ brain and adipose tissue is resulted from sleep deprivation (Davis et al. 2007; Gharib et al. 2012). In another study, four miRNAs in mice which are independent of corticosterone levels are changed because of sleep deprivation (Mongrain et al. 2010). Other research shows that intraventricular and cortical injection of miRNAs/anti-miRNAs (inhibitors) into rat brain can alter sleep and electroencephalographic (EEG) slow wave activity (Davis et al. 2011, 2012). But for human sleep regulation and sleep disturbances, it has not been identified.

To explore the general profile of thousands of molecules in parallel in a disease of uncertain origin, different methods have been used to profile miRNAs expression, including Northern blotting with radio labeled probes, oligo-nucleotide microarrays, quantitative PCR-based amplification of precursor or mature miRNAs and so on. Recently one high-throughput technology named microarray is allowed for producing such orders of magnitude more data, which addresses the short nature of miRNAs and should be able to distinguish between miRNAs that differ by as little as a single nucleotide (Chen and Storey 2014; Alvarez-Mora et al. 2013). Based on result of high-throughput profiling, it is suggested that qRT PCR is still the gold standard for expression validation (Bardelle et al. 2015; Dedeoglu 2014; Qin et al. 2006).

In the present study we aimed to determine the microRNA profiling in peripheral blood samples from Narcolepsy patients and controls, and then validate the significant microRNA differences by qRT PCR which is still remained gold standard. Our main focus is on potential treatment options and novel therapeutic concepts which are in various stages of development. The abnormal microRNA expression may give insight into the pathophysiological mechanisms responsible for the symptoms of these sleep disorders, which is for better monitoring disease progress.

## Methods

### Patient characteristics, clinical features and peripheral blood harvest

As shown in Table 1, 20 patients diagnosed with narcolepsy were recruited for the initial screening of miRNAs in peripheral blood at the Shandong Provincial Hospital Affiliated to Shandong University, Shandong, China. Other 20 healthy controls without medical, neurological, or sleep abnormalities were recruited for the initial screening of miRNA by advertising for normal volunteers. According to International Classification of sleep disorders (ICSD), all patients are consistent with the clinical characteristics of Narcolepsy. Narcolepsy diagnosis was based on the following criteria: (1) excessive daytime sleepiness, (2) mean sleep latency <8 min, (3) cataplexy, (4) HLA-DQB1*06:02. Exclusion criteria were the presence of neurological, psychiatric or medical disorders. 20 Narcolepsy patients were free of antidepressants and stimulants 7–14 days before inclusion. Their hypersomnia history was obtained by a semi-structured interview based on the Stanford Sleep Questionnaire. All patients and volunteers provided consent for the use of their specimens in research, and this use was approved by the institute research ethics committee of the Shandong Provincial Hospital Affiliated to Shandong University.

**Table 1:** Demographic, clinical, sleep, and biological data of central hypersomnia patients and healthy controls in miRNA screen study

	Narcolepsy (N = 20)	Healthy controls (N = 20)
Gender (male), n (%)	8 (40 %)	10 (50 %)
Age, years	28.43 (± 6.25)	30.25 (± 5.89)
Body mass index (BMI)	25.70 (± 2.28)	21.20 (± 1.92)
Age of onset, years	19.76 (± 2.95)	–
Disease duration	12.30 (± 4.90)	–
Epworth sleepiness scale	19.00 (± 2.09)	–
Cataplexy, n (%)	18 (90 %)	–
Hypnagogic hallucinations, n (%)	18 (90 %)	–
Sleep paralysis, n (%)	20 (100 %)	–
Awakenings/night	5.93 (± 3.21)	–
Sleep latency, sec	296.08 (± 102.25)	–
PSG^a^—Total sleep time (TST), min	398.90 (± 80.21)	–
PSG^a^—Sleep efficiency, %	87.32 % (± 60.28)	–

^a^Polysomnography

### Total RNA isolation and reverse-transcription

Total RNA was harvested using Trizol (Invitrogen, CA, US) and an RNeasy Mini Kit (Qiagen, German) according to the manufacturer’s instructions. RNA quality was ascertained using an Agilent 2100 bioanalyzer (Agilent technologies). 1 μg of total RNA was reverse-transcribed and the product (11 μl) was pre-amplified using Megaplex PreAmp Primers and DBI Bestar® qPCR RT Kit (Applied Biosystems) in a 20 μl PCR reaction. The pre-amplification cycling conditions were 37 °C for 60 min and 98 °C for 10 min. The pre-amplified cDNA was diluted with 0.1 × TE (pH 8.0) to 10 μl and then 1 μl diluted cDNA was used in each plate for qRT PCR reactions.

### MiRNA microarray analysis

After RNA quan-tification using a Nanodrop 2000 spectrophotometer, the samples were labeled using the miRCURYHy3/Hy5 Power Labeling Kit and hybridized to the miRCURY LNA microRNA Array (v.11.0). The samples were hybridized using a hybridization station and the arrays were scanned with the Axon GenePix 4000B Microarray Scanner. The raw intensity of the image was read using GenePix Pro V6.0. The intensity of the green signal was calculated after background subtraction, and four replicated spots for each probe on the same slide were averaged. The Median Normalization Method was used to obtain ʻNormalized Data’ [Normalized Data = (foreground-background)/median]. The median was defined as the 50 % quantile of microRNA intensity that was >50 in all samples after background correc-tion. The statistical significance of the differentially expressed microRNA was analyzed using the Student’s t test.

### Quantitative RT-PCR of mature miRNAs

Real-time quantification was performed using an Applied Bio systems 7500 Sequence Detection system. The 20 µl PCR reaction included 1 µl RT product (1:5 dilution), 0.5 µl Universal reverse primer, 0.5 µl of sense primer, and 10 µl mix buffer (DBI Bestar® SybrGreen qPCR masterMix). The reactions were incubated in a 96-well optical plate at 94 °C for 2 min, followed by 40 cycles of 94 °C for 20 s, 58 °C for 20 s and 72 °C for 20 s. All reactions were run in triplicate. All primers used are listed in Table 2.

**Table 2:** Sequence of the primers used for validation of selected miRNAs

Gene	Primer sequence (5′–3′)
hsa-miR-1267	Forward: ACACTCCAGCTGGGCCTGTTGAAGTGT
hsa-miR-4309	Forward: ACACTCCAGCTGGGCTGGAGTCTAG
hsa-miR-554	Forward: ACACTCCAGCTGGGGCTAGTCCTGAC
hsa-miR-182-3p	Forward: ACACTCCAGCTGGGTGGTTCTAGACTTG
hsa-miR-4501	Forward: ACACTCCAGCTGGGTATGTGACCTCG
hsa-miR-1272	Forward: ACACTCCAGCTGGGGATGATGATGGCAGC
hsa-miR-1272	Forward: ACACTCCAGCTGGGAGGGGGAAAGT
hsa-miR-1272	Forward: ACACTCCAGCTGGGTCCCTGAGACC
hsa-miR-4522	Forward: ACACTCCAGCTGGGTGACTCTGCCTGT
hsa-miR-4522	Forward: ACACTCCAGCTGGGAACCCGTAGATC
hsa-miR-197-3p	Forward: ACACTCCAGCTGGGTTCACCACCTTCT
hsa-miR-197-3p	Forward: ACACTCCAGCTGGGTTGTACATGGTA
U6	Forward: CTCGCTTCGGCAGCACA
U6	Reverse: AACGCTTCACGAATTTGCGT
All	Reverse: CTCAACTGGTGTCGTGGA

### Data analysis

MicroRNA microarray data were analyzed by LC Sciences by subtracting the background and normalizing the signals using a locally-weighted regression filter by 5S rRNA, as described previously. A miRNA was listed as detectable when it met at least three criteria: (1) signal intensity higher than 3 × the background standard deviation; (2) spot coefficient of variation (CV) <0.5, in which CV was calculated as (standard deviation)/(signal intensity); and (3) at least 50 % of the repeated probes had a signal 3-times higher than background standard deviation. The miRNA microarray data used the total gene signal, which was proportional to the total number of targets bound by the probes targeting each miRNA. Differentially expressed signals were determine by t-test with *p* < 0.01. To compare qPCR-array and microarray assays, the log2 of microarray signals was used.

qRT PCR assay was used to determine the changes in the expression of the target miRNAs in peripheral blood samples. The change in amplification was normalized to the expression of the U6 RNA. The fold change in expression was calculated for each sample using 2-ΔΔCt, where ΔΔCT = (Ct target gene-CtU6) narcolepsy–(Ct target gene-CtU6) control. A2-ΔΔCt >1.5 or <0.67 was considered differentially expressed miRNAs.

## Results

### Differentially expressed microRNAs in narcolepsy patients

**Fig. 1:**
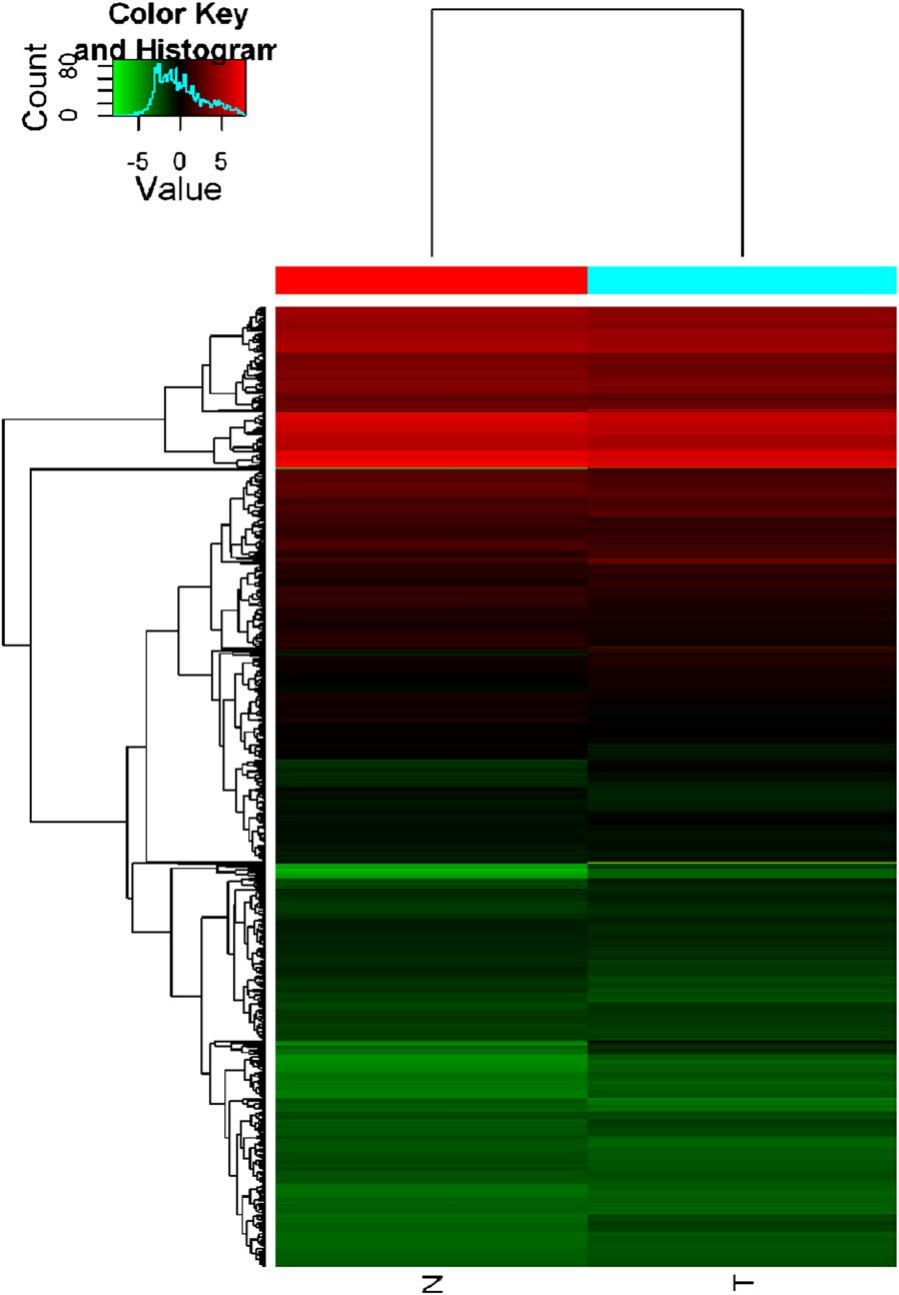
The cluster analysis of miRNAs expression in peripheral blood samples screened by miRNA microarray. Heat map: the horizontal for different hsa-miRNAs, two columns for different cohorts. *Red* for up-regulated hsa-miRNAs, *green* for down-regulated hsa-miRNAs, *black* for no differentially expressed hsa-miRNAs. *Color shade* represents the intensity of fluorescence and reflects the level of hsa-miRNAs expression. The scheme indicated that the clustering properties of gene expression in narcolepsy patients were obvious. *N* normal healthy controls; *T* tested narcolepsy patients

miRNA expression profiling studies were conducted using the miR-CURY LNA microRNA Array (v. 11.0), which contains probes human miRNAs. A total of 2805 miRNAs were identified. Among these, 105 miRNAs with a more than twofold change were differentially expressed between the normal peripheral blood and GC, and 23 miRNAs were significantly low expressed (Fig. 1).

### Validation of microRNA microarray results in clinical samples by qRT PCR

**Fig. 2:**
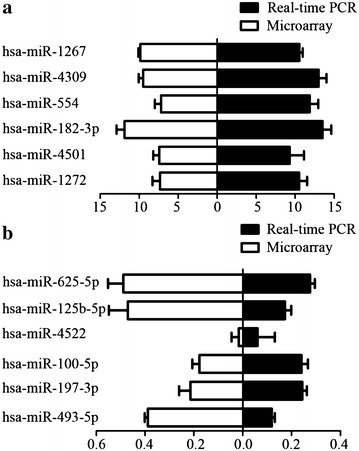
12 differential hsa-miRNAs related to narcolepsy sensitivity between cohort narcolepsy patients and healthy controls were screened and identified by real-time PCR. **a** High level of six hsa-miRNAs randomly selected for the validation of expression level by qRT-PCR is consistent with the result from miRNA microarray; **b** Low level of six hsa-miRNAs randomly selected for the validation of expression level by real-time RT-PCR is consistent with the result from miRNA microarray

**Fig. 3:**
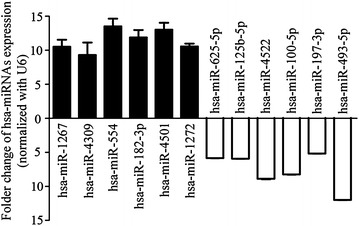
Validation of 12 hsa-miRNAs using qRT PCR shows hsa-mir-1267, hsa-miR-4309, hsa-miR-554, hsa-miR-1272, hsa-miR-4501, hsa-miR-182-3p were up-regulated and hsa-miR-625-5p, hsa-miR-100-5p, hsa-miR-125b-5p, hsa-miR-197-3p, hsa-miR-4522, hsa-miR-493-5p were down-regulated in each of the peripheral blood samples from narcolepsy patients. *Black* folder change of narcolepsy patients/control, *white* folder change of control/narcolepsy patients

In order to confirm the results obtained from the miRNA microarray, the expression of 12 miRNAs were analyzed by qRT PCR in the samples analyzed on the microarray. Consistent with the results from the microRNA microarray (Fig. 2a, b), hsa-mir-1267, hsa-miR-4309, hsa-miR-554, hsa-miR-1272, hsa-miR-4501, hsa-miR-182-3p were up-regulated and hsa-miR-625-5p, hsa-miR-100-5p, hsa-miR-125b-5p, hsa-miR-197-3p, hsa-miR-4522, hsa-miR-493-5p were down-regulated in each of the peripheral blood samples (Fig. 3).

## Discussion

Recent data has dem-onstrated the efficacy of miRNAs in the prevention, prediction and treat-ment of patients in a lot of diseases (Summerer et al. 2015; Tahara et al. 2013; Siemelink et al. 2013; Hubaux et al. 2012; Liu et al. 2009). We believe miRNAs in narcolepsy might act as molecular predictors of disease development and management outcome. So only one molecular-based treat-ment decision in these patients may be prob-lematic, not only at diagnosis but also at pro-gression, to detect some sensitive gene mutations. This study shows for the first time that it is possible to detect differences in a whole miRNAs profiling via microarray in plasma from patients with sleep disorders (Holm et al. 2014). In our research, we analyzed the expression of 2805 hsa-miRNAs in peripheral blood samples for the association between important mRNA degradation and clinicopathological data after miRNA microarray and validation experi-ments in vitro were performed. Microarray results showed that plasma levels of the 105 hsa-miRNAs highly expressed and 23 hsa-miRNAs had low expression which suggested that they may be asso-ciated with narcolepsy status. Among these miRNAs with significant change, 12 hsa-miRNAs were validated by qRT PCR which showed that hsa-mir-1267, hsa-miR-4309, hsa-miR-554, hsa-miR-1272, hsa-miR-4501, hsa-miR-182-3p had significantly high expression and hsa-miR-625-5p, hsa-miR-100-5p, hsa-miR-125b-5p, hsa-miR-197-3p, hsa-miR-4522, hsa-miR-493-5p had significantly low expression in each of the peripheral blood samples. Our results suggested a potential therapeutic target of miRNAs for narcolepsy.

As we know, usually 3′ UTR of genes are targeted by miRNA resulting in a significant reduction of full-length protein. So miRNAs with remarkable change in *silico* analysis may target some important genes which play an important role in narcolepsy (Holm et al. 2014). Some *silico* analysis implied that some important molecular signaling pathway such as IP3 pathway, IGF1R pathway, cAMP pathway may be associated with cognitive enhancement in animal models of brain disease (Jellen et al. 2015). Based on previous research, it is suggested that *TAC1*, *PENK* and *SOCS2* might be intimately connected with the excessive daytime sleepiness not only in dogs, but also in other species, possibly including humans (Lindberg et al. 2007). Other studies revealed that the diagnosis of narcolepsy but not *CPT1B* expression level was associated with abnormally and significantly low acylcarnitine levels (Miyagawa et al. 2011). Recently a new study discovered that HLA risk loci and protective variants for narcolepsy, which were independent of the well-established HLA-DQ effects in narcolepsy (Ollila et al. 2015). And function of *tbx3* in neurons may be effective in populations with sleep abnormalities (Eriksson and Mignot 2009). Further studies are required to clarify the relationship between miRNAs we screened out and those important genes during narcolepsy.

In conclusion, we have identified 12 aberrant miRNAs (hsa-mir-1267, hsa-miR-4309, hsa-miR-554, hsa-miR-1272, hsa-miR-4501, hsa-miR-182-3p, hsa-miR-625-5p, hsa-miR-100-5p, hsa-miR-125b-5p, hsa-miR-197-3p, hsa-miR-4522, hsa-miR-493-5p) in plasma from patients with sleep disorder. According to previous research, some miRNAs we screened out were involved in some other functions. For example, miR-625-5p may act as one of potential mediators of hypoxic response in soft tissue sarcomas (STS); miR-100-5p appeared to be important to regulation some gene expression during GC reaction. However, the biological significance of them in narcolepsy remains unclear. It is proposed that exploring the molecular mechanism of these miRNAs and their targets might increase our understanding of the pathogenesis of narcolepsy. Thus our results provide a new perspective on investigating sleep disorders, and offer a new approach to identifying relevant pathways in the pathogenesis of narcolepsy.
